# Expanding the use of ethanol as a feedstock for cell-free synthetic biochemistry by implementing acetyl-CoA and ATP generating pathways

**DOI:** 10.1038/s41598-022-11653-3

**Published:** 2022-05-11

**Authors:** Hongjiang Liu, Mark A. Arbing, James U. Bowie

**Affiliations:** Department of Chemistry and Biochemistry, Molecular Biology Institute, UCLA-DOE Institute of Genomics and Proteomics, University of California Los Angeles, Boyer Hall, 611 Charles E. Young Dr. E, Los Angeles, CA 90095-1570 USA

**Keywords:** Biocatalysis, Enzymes, Metabolic engineering

## Abstract

Ethanol is a widely available carbon compound that can be increasingly produced with a net negative carbon balance. Carbon-negative ethanol might therefore provide a feedstock for building a wider range of sustainable chemicals. Here we show how ethanol can be converted with a cell free system into acetyl-CoA, a central precursor for myriad biochemicals, and how we can use the energy stored in ethanol to generate ATP, another key molecule important for powering biochemical pathways. The ATP generator produces acetone as a value-added side product. Our ATP generator reached titers of 27 ± 6 mM ATP and 59 ± 15 mM acetone with maximum ATP synthesis rate of 2.8 ± 0.6 mM/h and acetone of 7.8 ± 0.8 mM/h. We illustrated how the ATP generating module can power cell-free biochemical pathways by converting mevalonate into isoprenol at a titer of 12.5 ± 0.8 mM and a maximum productivity of 1.0 ± 0.05 mM/h. These proof-of-principle demonstrations may ultimately find their way to the manufacture of diverse chemicals from ethanol and other simple carbon compounds.

## Introduction

To combat global warming, it is estimated that we need to move to a net carbon negative impact by year 2050^1^. Petroleum forms the basis for most of the chemical compounds used industrially, thereby contributing to greenhouse gas emissions^2^. If these petroleum-based chemicals could be built from carbon recycled from the atmosphere it could greatly reduce our carbon footprint.

**Figure 1:**
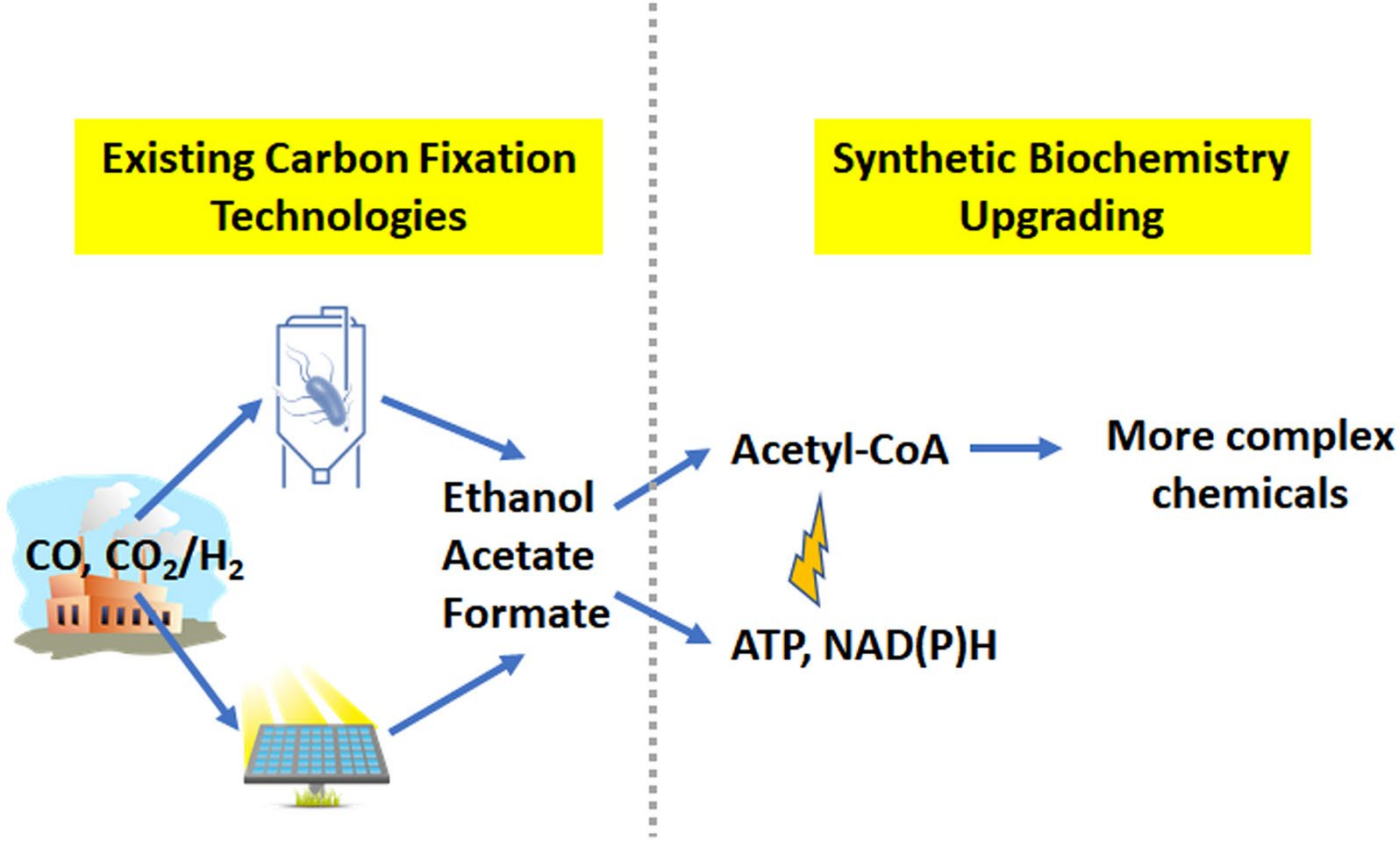
A possible path to a carbon negative chemical industry. Carbon is first fixed from the atmosphere into simple carbon compounds like ethanol and acetate using acetogens or electrochemistry. Cell-free synthetic biochemistry can then be used to make more complex chemicals used directly or as building block carbon chemicals. These building block carbon chemicals could then be used to build still more complex chemicals using existing or new synthetic methods. Key enabling technologies for the application of synthetic biochemistry are methods to generate a central biochemical building block, acetyl-CoA, and the biochemical energy carrier, ATP.

A potential approach for generating diverse carbon-negative chemicals is illustrated in Fig. 1. The concept starts with simple carbon compounds that can be produced with carbon fixed from the atmosphere. Advances in microbiology, metabolic engineering and electrochemistry have made possible the carbon negative production of simple one and two carbon compounds. In particular, acetogens can efficiently convert flue gas into ethanol and acetate and there are several commercial plants in development^3–6^. A second development is advances in electrochemical carbon capture which can convert CO_2_ into small carbon compounds like formate and ethanol with increasing efficiency^7–10^. To the extent that the electrical power is derived from the sun or nuclear plants, electrochemistry provides another carbon negative process for making simple carbon compounds. Effective ways to upgrade these simple molecules into more complex chemicals could therefore potentially form the basis for a carbon negative chemical industry. To realize this vision of a carbon negative economy, it will be necessary to develop effective, sustainable methods for converting simple carbon compounds into more diverse chemicals^11^. The upgraded chemicals could be used directly or employed as precursors for building additional chemical diversity, thereby replacing myriad petroleum derived chemicals with carbon negative chemicals.

While it may be possible to use engineered microbes to upgrade simple carbon compounds like ethanol acetate and formate, an enzymatic cell-free conversion could provide more efficient methods for complexifying the simple fixed carbon compounds. A cell-free, synthetic biochemistry approach could potentially provide many advantages over cell-based conversions in yield, productivity and titers (reviewed in^12^), but will need many advances before it is possible to employ enzymes on a scale needed for commodity chemical manufacturing. The first step is to develop pathways that could become viable for cell-free production.

Some initial steps have been made to employ ethanol as a building block chemical for cell-free synthesis. Zhang et al. developed a system to enzymatically convert ethanol into 2,3-butanediol and 2-butanol^13^. We developed a simple system for upgrading ethanol into 1,3 butanediol^14^. In both of these efforts, the pathways run through acetaldehyde which is then fused to make four carbon molecules. Reducing equivalent power in the form of NAD(P)H is supplied by formate oxidation. None of these pathways utilize the central biochemical intermediate acetyl-CoA or key energy carrier, ATP.

To increase the diversity of molecules that can be made biochemically from ethanol, it will be important to be able to produce acetyl-CoA from ethanol as acetyl-CoA provides a gateway to myriad biochemical pathways. We also need a way to generate the ATP required for many pathways. Here we show that acetyl-CoA can be generated straightforwardly from ethanol in a cell-free pathway and show how to generate ATP for powering cell-free systems. These pathways add to the toolbox for developing a sustainable chemical industry based on carbon-negative building blocks.

## Results and discussion

### Converting ethanol to acetyl-CoA

**Figure 2:**
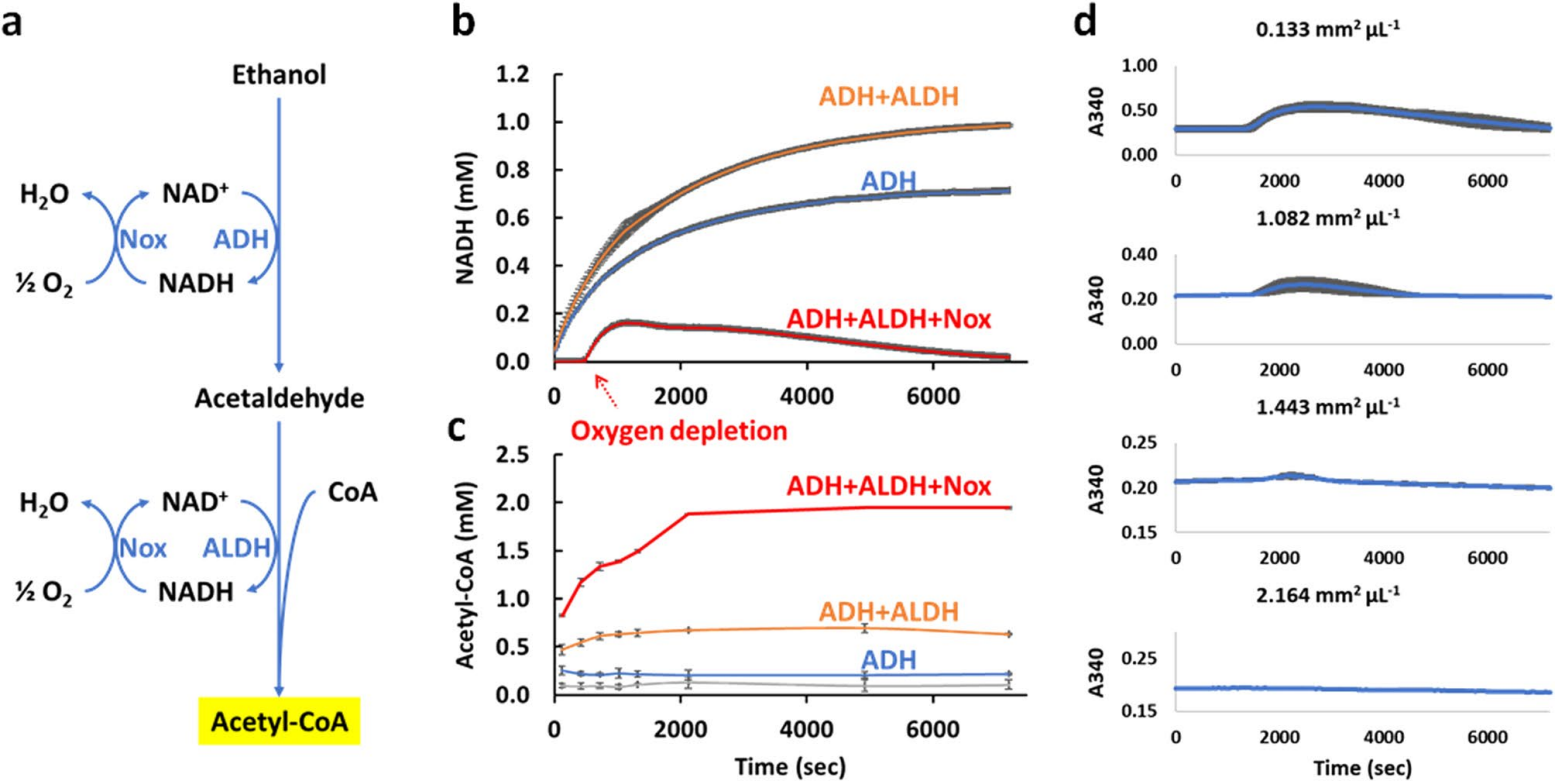
Ethanol to acetyl-CoA module. (**a**) Schematic of the pathway employed. Reoxidation of NADH, catalyzed by Nox, maintains a concentration gradient to drive the otherwise thermodynamically unfavorable reaction. (**b**) NADH concentration over time with different enzyme combinations as indicated in the figure. (**c**) Acetyl-CoA concentration over time with different enzyme combinations as indicated in the figure. (**d**) The effect of surface area to volume on NADH concentrations over time, using the full system with ADH, ALDH and Nox. All assays experiments were performed in biological triplicates and the error bars reflect the standard deviation.

Our approach to converting ethanol into acetyl-CoA is shown in Fig. 2a. We first oxidize ethanol to acetaldehyde using alcohol dehydrogenase (ADH) and NAD^+^. Acetaldehyde is then converted to acetyl-CoA using aldehyde dehydrogenase (ALDH). The first oxidation step (ADH reaction) is a highly unfavorable thermodynamically^15^. While the second reaction (ALDH reaction) is favorable thermodynamically, the overall reaction is still unfavorable by + 14.5 ± 2.4 kJ/mol at a 1 mM standard state^15^. Thus, we need a mechanism to drive the overall reaction forward to produce acetyl-CoA. To accomplish this goal, we introduce an enzyme NADH oxidase (Nox) that re-oxidizes NADH back to NAD^+^, which provides a large ∆G° driving force of − 433.5 ± 6.4 kJ/mol. The Nox-catalyzed reaction can thereby maintain a large NAD^+^/NADH gradient to drive the conversion of ethanol to acetyl-CoA.

To test the system for producing acetyl-CoA from ethanol, we built reactions with ADH alone; with ADH and ALDH; and a full system with ADH, ALDH and Nox. We used 2 mM CoA so the maximum possible production of acetyl-CoA would be 2 mM. As shown in Fig. 2b,c, ADH alone (blue traces) oxidizes ethanol, generating NADH as expected, but does not produce acetyl-CoA. However, when ADH was coupled with ALDH (orange traces), the NADH production increased, and some acetyl-CoA was produced. When the full-length acetyl-CoA production module was assembled (red traces), Nox rapidly reoxidized NADH back to NAD^+^ (Fig. 2b), allowing acetyl-CoA production up to 1.94 ± 0.01 mM (Fig. 2c), approaching the theoretical maximum. Thus, we were able to drive the reaction to near completion by the addition of Nox. The acetyl-CoA synthesis rate for the first 2000s was ~ 3.2 mM/h.

The changes in NADH concentration over time in the full system (ADH, ALDH, Nox) is complex as might be expected for an oxygen dependent reaction. As shown in (Fig. 2b red trace), the NADH concentration is initially undetectable, then increases to a maximum of ~ 0.1 mM at ~ 1000 s, then decreases gradually over time. We hypothesize that this behavior was due to oxygen levels in the reaction. In particular, approximately 0.2 mM oxygen is readily dissolved in water under standard state conditions^16^. So, upon production of ~ 0.2 mM NADH, the dissolved oxygen in solution would be depleted by the activity of Nox. Consistent with this view, the NADH concentration begins to increase after about 0.2 mM NADH is generated (compare to reactions without Nox). At that point the rate of re-oxidation of NADH would be limited by air/water diffusion. To test this hypothesis, we varied the surface area to volume ratio of the reactions by performing the reactions in different sized containers. As shown in Fig. 2d. The NADH concentration rise is more significant at smaller surface:volume ratios and the onset is sooner, while at larger surface:volume, the rise is mitigated. These results suggest that the rate of aeration is indeed a limiting factor after the initial dissolved oxygen is consumed.

### An ATP generating module to power cell-free synthesis

Economical cell-free systems must employ methods to recycle ATP^17^. It is not viable to simply supply the required ATP due to cost, and the buildup of ADP and phosphate will eventually poison the systems. Recycling can be done by using sacrificial substrates such as creatine phosphate or polyphosphate, but this approach does not solve the problem of waste product accumulation. Thus, if we want to create cell-free systems that can operate continuously for long periods of time, it is essential to develop ATP generation methods that avoid the buildup of waste products. We therefore wanted to test whether we could use the energy stored in ethanol develop a useful ATP generator for cell-free systems.

**Figure 3:**
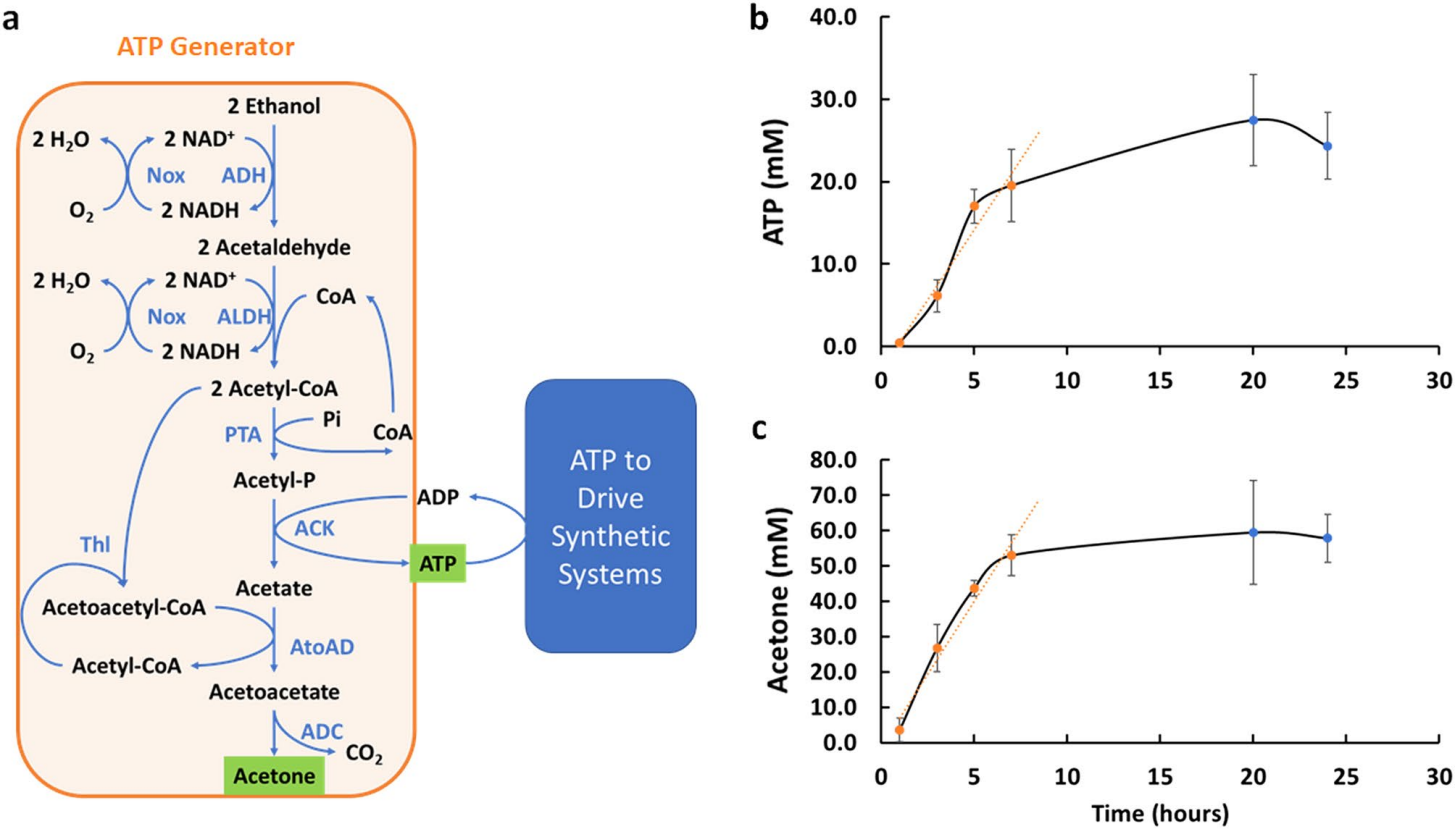
ATP generator module. (**a**) Schematic of the pathway employed. The input ethanol is converted to acetone, generating ATP in the process. The ATP can then be used separately to drive biochemical pathways. (**b**) The amount of ATP generated over time using the ATP generator module. (**c**) The amount of acetone generated over time using the ATP generator module. All assays experiments were performed in biological triplicates and the error bars reflect the standard deviation.

We designed a potential ATP Generator for the continuous generation of ATP shown in Fig. 3a. The production of acetyl-CoA from ethanol described above enables the production of ATP via the phosphotransacetylase (PTA) and acetate kinase (ACK) pathway. The PTA-ACK pathway generates the acetate, however, that would ultimately accumulate in the reaction solutions. To deal with the acetate buildup, we added three more enzymes to funnel the carbon into acetone. Acetone is volatile, which provides a means to remove it continuously from the reaction systems by gas stripping. Moreover, acetone is more valuable than ethanol or acetate, providing a value-added side product. To make acetone, we employ previously implemented pathways^18–20^ by first combining two acetyl-CoA to make acetoacetyl-CoA catalyzed by thiolase (Thl). The acetoacetyl moiety can be exchanged with acetate to produce acetyl-CoA and acetoacetate, catalyzed by acetate CoA-transferase (AtoAD). Acetoacetate can then be decarboxylated to acetone and CO_2_ via acetoacetate decarboxylase (ADC). The designed system will not only generate ATP for use in other reactions, it adds value by upgrading ethanol to a more valuable product, acetone.

To build the ATP generator, we divided the system up into two parts for testing. First, we wanted to examine our ability to implement ATP generation from ethanol via the PTA-ACK pathway. We therefore truncated the pathway at the ACK step, going from ethanol to acetate (pathway shown in Supplemental Fig. 1a). A seen in Supplemental Fig. 1a the pathway from ethanol to acetate generates ATP (quantified by glucose consumption in a coupled hexokinase reaction). Starting from an initial supply of 1 mM ADP, the minimally optimized system linearly generated/recycled 11.25 ± 0.01 mM ATP over 24 h, a rate of ~ 0.46 mM/h. We next wanted to test our ability to recycle the acetate into acetone, independent of ATP generation. We therefore built a system shown in Supplemental Fig. 2a, to convert ethanol into acetone using exogenously added acetate (rather than acetate generated via ATP production). As shown in Supplemental Fig. 2a, after 24 h of operation we observed 27 ± 1 mM acetone production from a system co-fed with ethanol and acetate. These results demonstrate that we had a viable set of enzymes in hand and we were ready to test the full system.

We then set out to build the full ATP Generation Module (Fig. 3). We first assembled the enzymes and cofactors making initial estimations as to appropriate enzyme levels and supplied the system with 2% ethanol (v/v). We then optimized the enzyme levels by either doubling the loading or halving the loading (see “Methods” for more details) until we saw no significant improvement in ATP and acetone titer in one day reactions. The final time courses of ATP and acetone production for the optimized system are shown in Fig. 3a,b. Both acetone and ATP levels increase rapidly for the first 7 h and then plateau, reaching titers of 27 ± 6 mM ATP and 59 ± 15 mM acetone. In the first 7 h, the ATP synthesis rate is 2.8 ± 0.6 mM/h and acetone is 7.8 ± 0.8 mM/h.

In theory, the amount of ATP produced and the amount of acetone produced should match, but we saw roughly two times as much acetone as ATP. We believe the discrepancy is due to depletion of ATP by contaminating ATPases, perhaps in the form of adenylate kinase that is heat stable and previously found to contaminate cell-free reactions^21^. We assayed for ATPase activity in the enzyme mixture and found that the enzyme mixture indeed contained 1.36 ± 0.008 mM/h ATPase. Efforts to reduce or eliminate ATPase contamination are ongoing, but is a difficult challenge for such a large collection of enzymes. In the current effort, it reduces efficiency of ATP production from ethanol, but does not prevent us from moving forward with testing the use of our ATP generator for powering other biosynthetic systems.

### Isoprenol production

Having demonstrated the ability to generate ATP and upcycle acetate waste to acetone, we sought to test the ability of the system to power the ATP-requiring phase of isoprenoid biosynthesis by making isoprenol from mevalonate (MVA). Isoprenoids comprise tens of thousands of molecules, many of which are employed in foods cosmetics, pharmaceuticals ^22^, and may provide useful biofuels^23^. MVA is being developed as a potential feedstock^24^ for high value isoprenoid production or isoprenol can also be used as a precursor for more complex isoprenoid biosynthesis^25–27^. Isoprenol has therefore been a target of metabolic engineering efforts, with Zheng et al.^28^ achieving ~ 15 mM isoprenol and George et al.^29^ obtaining ~ 26 mM and Kang et al.^30^ in a semi-scaled batch system obtaining ~ 125 mM in engineered *E. coli* cells.

**Figure 4:**
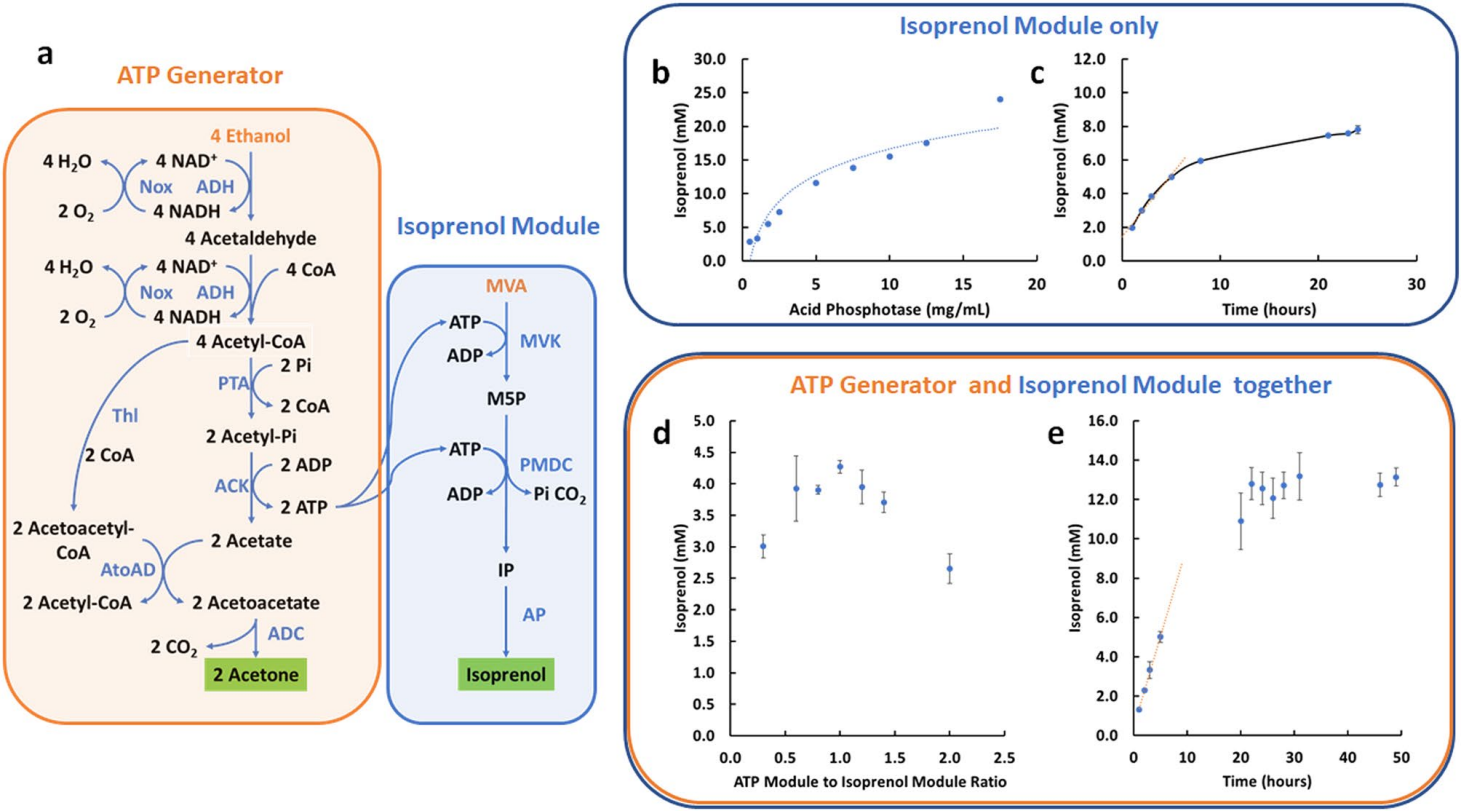
Coupling the ATP generator module to power isoprenol biosynthesis from mevalonate. (**a**) Schematic of the pathway employed. The ATP Generator Module supplies ATP to the two kinases required for the Isoprenol Module. (**b**) The AP amount limits the Isoprenol Module rate. The graph shows the amount of isoprenol produced by the isolated Isoprenol Module overnight as a function of the amount of AP added. To test the Isoprenol Module in isolation, ATP was supplied directly to eliminate the need for the ATP Generator Module. (**c**) Isoprenol production over time by the isolated Isoprenol Module. The AP concentration was set at 7 g/L. ATP was supplied to eliminate the need for the ATP Generator Module. (**d**) Optimizing the ratio of optimized ATP Generator Module enzyme concentrations to Isoprenol Module enzyme concentrations. The amount of isoprenol produced by the varying ratios after 5 h is shown as a measure of initial rate. The best ratio was found to be 1:1. (**e**) Isoprenol production over time using the full system shown in panel a and a 1:1 ratio of ATP Generator Module to Isoprenol Module enzymes. All assays experiments were performed in biological triplicates and the error bars reflect the standard deviation.

We designed an Isoprenol Module shown in Fig. 4a (blue box). Inspired by Kang et al.^31^, we decided to construct a minimal three step MVA to isoprenol pathway. First, MVA is phosphorylated by MVA kinase (MVK), consuming 1 ATP. Then through an alternative activity of the ATP-dependent decarboxylase, pyrophosphate mevalonate decarboxylate (PMDC), mevalonate-5-phosphate (M5P) is directly decarboxylated to isoprenol phosphate, with the consumption of a second ATP. PMDC normally decarboxylates mevalonate pyrophosphate^32^ but has been shown to also catalyze decarboxylation of the monophosphate (Supplemental Fig. 3)^31^. Finally, the isoprenol phosphate is hydrolyzed to isoprenol via an acid phosphatase (AP). In the designed system, the two ATPs required for the Isoprenol Module are supplied by the ATP Generator Module described above.

We first worked to optimize the Isoprenol Module (MVA to isoprenol conversion) by supplying ATP directly rather than from the ATP Generator Module. From initial guesses at enzyme levels, we varied the enzyme concentration by doubling or halving each enzyme individually and measured the amount produced after 16 h (See Supplemental Fig. 4 for details). The only enzyme concentration that significantly affected the titer was the Acid Phosphatase (AP). The other enzymes show minimal effects on titer when either doubled or halved. Hence, we worked to optimize the AP concentration as the AP dominates the total enzyme loading. As shown in Fig. 4b, the amount of isoprenol produced at a fixed time increases with AP concentration even up to 17 g/L, although the increase is less dramatic above 7 g/L. To keep the total enzyme loading in a reasonable range, however, we decided to fix AP usage at 7 g/L. A time course of isoprenol production with the Isoprenol Module alone at 7 g/L AP is shown in Fig. 4c. We obtained 7.8 ± 0.2 mM of isoprenol after 24 h of reaciton. The rate of production in the first 5 h was ~ 0.73 mM/h.

Clearly the AP enzyme specific activity will need improvement going forward. To our knowledge, no highly specific and efficient AP has yet been identified for the hydrolysis of isoprenol phosphate^28^. In metabolically engineered *E. coli,* the isoprenol phosphate hydrolysis reaction is apparently catalyzed by several different endogenous phosphatases that need further investigation^28^.

With semi-optimized ATP Generator (above) and Isoprenol modules in hand, we sought to put the entire system together (Fig. 4a). A full optimization of 11 enzymes is a major effort that is not warranted at this proof-of-principle stage so we simply varied the concentration of each module as a unit. The enzyme concentrations employed after partial optimization of each module separately we refer to as 1x. The maximum 1× rate we obtained for the ATP Generator Module was ~ 2.8 mM/h, while the 1× rate for Isoprenol Module was ~ 0.73 mM/h, corresponding to an ATP consumption rate of ~ 1.4 mM/h. Thus, in theory, the optimal rate should be obtained with a 1:2 ATP Generator Module to Isoprenol Module. As seen in Fig. 4d, we varied the ATP Generator Module enzyme concentrations from 0.2× to 2× relative to the Isoprenol Module enzyme concentrations and measured the amount of isoprenol production after 5 h. We found similar titers in a broad range of 0.6: to 1.4:1 module ratios, but the best ratio was 1:1, providing a titer after 5 h of 4.3 ± 0.1 isoprenol (compared to 3.9 ± 0.5 mM at 0.6:1, near the predicted optimum). We hypothesized that the full system with added enzymes leads to additional ATPase activity so that more ATP needs to be generated than anticipated. Indeed, we found that both the ATP Generator Module and the Isoprenol Module contain substantial ATPase background (the ATP Generator Module enzyme mixture contains 1.36 ± 0.008 mM/h ATPase while Isoprenol Module enzyme mixture contains 0.84 ± 0.02 mM/h ATPase at 1X enzyme concentrations). Given the loading ratio of both components, the addition of the Isoprenol Module will introduce ~ 47% more ATPase activity, suggesting an explanation for why we needed to run the ATP generator richer than predicted. Using the optimized 1:1 ratio, we monitored isoprenol production over time. As shown in Fig. 4e, the maximum titer is reached after a 24-h reaction, reaching 12.6 ± 0.8 mM of isoprenol with an estimated maximum rate of production of 8.5 mM/h during the first 5 h of reaction.

## Conclusion

In this work we have demonstrated that it is possible to efficiently convert ethanol to acetyl-CoA, opening up diverse avenues for upgrading ethanol to more complex molecules. As one application we upgraded ethanol to more valuable acetone via an acetyl-CoA intermediate. We also demonstrated a method for transferring the energy in ethanol into the biologically versatile ATP molecule, with an acetone side product. Acetone is volatile and can therefore be readily extracted continuously, so the pathway can provide a way to provide ATP power to cell free systems without building up waste products. Unlike traditional ATP recycle methods that rely on sacrificial substrates such as polyphosphate or creatine phosphate, the pathway avoids buildup of phosphate waste^17^. In this case, our ATP generator can recycle somewhere between 27 ± 6 mM ATP to 59 ± 15 mM ATP in 24 h of operation. We implemented a simple application of this concept by using the ATP Generator Module to drive the production of isoprenol from mevalonate.

The pathways described here expands the potential use of ethanol as a feedstock molecule for the generation of carbon negative chemicals. Clearly much work is needed for practical use of these pathways, however. Most notably, the ATP Generator Module will need to operate sustainably for much longer periods of time. We have not investigated why ATP generation stops, but enzyme stability is a common cause of reaction cessation in cell-free systems^14,33^. Furthermore, in larger scale implementation, volatile products either needed to be removed though gas stripping^34^ or solvent extraction^35^ and both methods place extra requirements on enzyme stability. Indeed, improving stability will be a critical element of further optimization and potential scaling demonstration. ATPases are common contaminants that complicate the setup of cell free systems and reduce efficiency. Extensive purification to remove ATPases will not be tractable for large scale cell-free manufacturing, so we need to develop simple methods for removing ATPase contamination. For example, the Honda group developed an *E. coli* strain with a temperature sensitive adenylate kinase, allowing elimination of adenylate kinase activity by a simple heat treatment^21^. A similar approach may be effective for removing other ATPases in *E. coli* extracts. Another major factor that will require additional developments is cofactor costs. We will need to develop methods for making cofactors more cheaply or employ cheaper cofactor analogs^36–38^. CoA is particular problematic in this regard so lowering CoA costs would be important enabling technology for cell-free manufacturing and therefore should be a focus of research in cell-free systems^21^.

## Materials and methods

### Molecular cloning

All genes except AtoAD were codon optimized for *E. coli* using Twist Biosciences (South San Francisco, CA) online suite (For DNA sequences please see “Supplemental Information”). All genes except AtoAD were synthesized and cloned by Twist Bioscience into the Nde1-Xho1 site of pET28b plasmid. The ADC clone was further manipulated to remove the N-terminal HisTag. The AtoAD complex is encoded within the genome of *E. coli* DH5α by a bicistronic operon in which the stop codon of AtoD overlaps with the start codon of the AtoA gene. The AtoAD bicistron was PCR amplified from *E. coli* DH5α genomic DNA using the primers 5′-AACCTGTATTTCCAGAGTATGAAAACAAAATTGATGACATTAC-3′ and 5′-GTGATGGTGATGGTGATGAGTTAAATCACCCCGTTGCGTATTC-3′. The PCR product was purified and cloned, by Gibson Assembly, into the pMAPLe3 expression vector^39^ which appends a hexahistidine tag to the C-terminus of the gene product (AtoA). Sequences were verified (Genewiz (now Azenta, Chelmsford, MA)) and used to transform *E. coli* Bl21 (DE3) Gold and individual colonies selected. The transformed strains were stored frozen in culture medium with 30% glycerol at − 80 °C.

### Protein purification

For enzyme production and purification, frozen stocks were used to inoculate 1 L auto-induction media containing 50 mg/L kanamycin. The auto-induction media was prepared by adding 0.5 g glucose and 2 g lactose mixture into Miller’s formula Luria–Bertani medium and then autoclaved for 30 min. The cells were grown for approximately 20 h at 37 °C and the cells collected by centrifugation at 3720×*g* for 30 min. The cell pellets were resuspended and incubated in 20 mL Hypotonic Thermolysis Buffer (50 mM NaCl, 20 mM Tris–HCl pH 7.5) with 2.5 mg chicken egg-white lysozyme (Sigma-Aldrich, St. Louis, MO) for 10 min. Then, the resuspensions (except AtoAD and Nox) were heated to 60 °C for 30 min. Since AtoAD and Nox are not thermostable, AtoAD and Nox suspensions were lysed with an Emulsiflex homogenizer at 10,000 bars, substituting for the heat step. All the cell lysates were then incubated with 2500 units benzonase nuclease (Sigma-Aldrich) and centrifuged at 24,465×*g* for 30 min.

The resulting clear supernatants were further processed by Ni–NTA chromatography (except ADC, which did not have a 6xHis tag). First, the clear lysates were incubated with 3 mL (bed volume) of Ni–NTA Superflow (Qiagen (Maryland, US) for 30 min at 4 °C. Then, the resulting mixtures were transferred to a gravity flow column and washed with 2 × 10 mL Hypotonic Thermolysis Buffer followed by 2 × 10 mL Wash Buffer (300 mM NaCl, 50 mM Tris–HCl pH 7.5, 5 mM imidazole). After washing, the enzymes bound to the Ni–NTA bed were eluted with Elution Buffer (150 mM NaCl, 50 mM Tris–HCl pH 7.5, 250 mM imidazole, 10% glycerol).

For the tagless ADC, a clear, heat-treated lysate was prepared as described above, and then concentrated with a 30 KD cutoff concentrator (Amicon Ultra) to below 5 mL. The concentrated lysate was then dialyzed into Storage Buffer (150 NaCl, 50 mM Tris–HCl pH = 7.5 and 10% glycerol).

Protein concentrations were measured by absorbance at 280 nm and purity evaluated SDS-PAGE. Visually, the purified Nox enzyme was brilliantly yellow, while the rest were colorless. For long term storage, all enzymes were flash-frozen in Elution Buffer with liquid nitrogen and stored in − 80 °C.

### Ethanol oxidation to acetyl-CoA

To set up the testing reactions, 1 mM NAD^+^, 1 mM CoA and 100 mM ethanol were mixed in General Buffer (GB, consists of 10 mM KCl, 50 mM NaCl, 10 mM MgCl_2_ and 100 mM Tris–HCl buffer at pH 7.5). Different enzymes combinations were added to start the reaction: (1) 0.01 g/L ADH; (2) 0.01 g/L ADH plus 0.02 g/L ALDH or (3) 0.01 g/L ADH, 0.02 g/L ALDH and 0.08 g/L Nox at a final reaction volume of 200 μL. 340 nm absorbance (A340) changes were recorded with a Molecular Device SpectraMax M5 96-well plate reader.

Acetyl-CoA concentrations were measured by a free-thiol assay using Ellman’s Reagent (5,5-dithio-bis-(2-nitrobenzoic acid), DTNB). First, a fresh Ellman’s stock reagent was made containing 1 g/L of DTNB in Ellman’s Buffer (1 mM EDTA and 100 mM sodium phosphate at pH 8). The free-thiol measurements were performed by mixing 50 μL samples with 10 μL Ellman’s reagent and 140 μL Ellman’s Buffer. Following incubation at room temperature for 15 min, the absorbance at 412 nm was measured and compared to a calibration curve. The calibration curve was prepared using authentic trilithium CoA (Sigma-Aldrich, St. Louis, MO).

To observe the relationship between oxygen availability and NADH levels, the same ADH + ALDH + Nox reactions were set up in various multiwell plates with distinct surface to volume ratios plates.

### Acetone production and ATP production

To test acetone production with ethanol and acetate as co-feed we used 4 mM of NAD^+^, 4 mM CoA, 1% ethanol and 50 mM acetate (pH 7.4) in GB. To start the reaction, an enzyme mixture was added to final concentrations of 0.13 g/L of ADH, 0.85 g/L of ALDH, 0.51 g/L of Nox, 0.21 g/L of Thl, 0.5 g/L of AtoAD and 2.65 g/L of ADC. The final reaction volume was 200 μL. To ensure sufficient oxygen while preserving the volatile components, the reactions were performed in 12 mL glass screw top vials. The reactions were performed at 37 °C and incubated the same manner as before. After reaction, the solutions were brought back to room temperature and 400 μL of phenetole was quickly added to the reaction. The mixture was then transferred to solvent resistant centrifuge tubes and vigorously vortexed and spun down in a centrifuge. The organic phase was collected for Gas-Chromatography with Flame Ionization Detection (GC-FID) with a Fisher Scientific Trace 1310 system. The titer was extrapolated from a standard curve of analytical acetone that has been treated the same way with enzyme reaction in buffer and shaking.

To test ATP production with ethanol as the sole-feed we used 4 mM of NAD^+^, 4 mM CoA, 1 mM ADP, 20 mM Na-Pi (pH 7.4), 1% ethanol and 50 mM Glucose in GB. To start the reactions a mixture of enzymes was added to final concentrations of 0.13 g/L of ADH, 0.85 g/L of ALDH, 0.51 g/L of Nox, 0.21 g/L of Thl, 0.14 g/L of PTA, 0.045 g/L of ACK and 0.2 g/L of hexokinase. The final volume was 200 μl. The reactions were measured individually at various time points ranging from 1 to 24 h. To measure ATP generation, we utilized a reporter platform by converting glucose and ATP to glucose-1-phosphate through hexokinase. This single step reaction recycles ADP/ATP while consuming free reducing sugar. The amount of remaining free reducing sugar can be measured with a glucose assay (adapted from Sigma-Aldrich, St. Louis, MO). Simply, Glucose Assay Buffer was freshly prepared, consisting of 60 mM potassium phosphate (pH 5.9), 0.012% 4-amino-antipyrine, 0.024% *N*-Ethyl-*N*-(2-hydroxy-3-sulfopropyl)-m-toluidine (EHSPT), 0.012 mM FAD, 1.2 mM EDTA, 16 mM MgCl2, trace amount of peroxidase (Goldbio, St Louis, MO) and glucose oxidase (Calbiochem, San Diego, CA). The assay mixture was made fresh each time before use and the glucose calibration curve was plotted freshly along with a new mixture right before use. 5 μL of assay samples were mixed with 95 μL freshly made Glucose Assay Buffer, incubated at 37 °C for 30 min and the absorbance 550 nm measured. The absorbance response was then compared to a standard curve prepared with glucose.

For the reactions combining ATP production and acetone production into a continuous co-production process we used 4 mM of NAD^+^, 4 mM CoA, 1 mM ADP, 20 mM sodium phosphate (pH 7.4), 1% ethanol and 50 mM Glucose in GB. To start the reactions, an enzyme mixture was added to make final concentrations of 0.13 g/L of ADH, 0.85 g/L of ALDH, 0.51 g/L of Nox, 0.44 g/L of Thl, 0.14 g/L of PTA, 0.045 g/L of ACK, 0.5 g/L of AtoAD and 2.65 g/L of ADC and 0.2 g/L of hexokinase. Then the samples were individually measured at various time points with both the Glucose Assay to measure ATP regeneration and by GC-FID to measure acetone co-production as described above.

### ATP acetone co-production optimization

To optimize the performance of ATP production, we either doubled or halved all components in the co-production scheme in every round of optimization. The best outcome conditions were combined to initiate the next round of optimization. This approach was repeated 3 times. The best conditions found were as follows: 4 mM of NAD^+^, 4 mM CoA, 1 mM ADP, 20 mM sodium phosphate (pH 7.4), 1% ethanol and 50 mM Glucose, 0.38 g/L of ADH, 0.85 g/L of ALDH, 0.51 g/L of Nox, 0.22 g/L of Thl, 0.14 g/L of PTA, 0.045 g/L of ACK, 0.25 g/L of AtoAD and 1.5 g/L of ADC and 0.2 g/L of hexokinase in GB.

### Isoprenol module optimization

To optimize the performance of MVA to isoprenol production, the MVA, PMDC, and Acid Phosphatase (MP Biomedicals, Irvine, CA) were either doubled or halved (high and low conditions). After determining that the rate was limited by the AP concentration, AP usage was gradually increased from 0.5 to 17.5 g/L. Considering the large enzyme load and volume constraints, we set the AP level at 7 g/L acid. Since the total enzyme loading is so dominated by the AP concentration, we did not further adjust the MVA and PMDC concentrations. The final enzyme concentrations considered to be broadly optimized were: 0.1 g/L MVK, 0.4 g/L PMDC and 7 g/L Acid Phosphatase.

### Full system optimization

To optimize the full-length system, the system was divided into two sub-systems: the ATP Generator Module and the Isoprenol Module. Using the optimized concentrations described above, we define a 1:1 ratio as one part of the optimized isoprenol module to one part of the optimized ATP module. We tested the mixture with ratios of the ATP Generator Module to Isoprenol Module ranging from 0.2 to 2.0 with the total volume fixed. All reactions were allowed to run for 5 h to obtain a rough estimation of initial rates, and assayed for isoprenol levels on GC-FID. The best condition is as follows: 4 mM NAD^+^, 4 mM CoA, 4 mM ADP, 20 mM sodium phosphate (pH 7.4), 2% ethanol, 0.38 g/L of ADH, 0.85 g/L of ALDH, 0.51 g/L of Nox, 0.22 g/L of Thl, 0.14 g/L of PTA, 0.045 g/L of ACK, 0.25 g/L of AtoAD and 0.5 g/L of ADC, 0.1 g/L MVK, 0.4 g/L PMDC, 7 g/L Acid Phosphatase, buffered in 1× General Buffer.

The reactions were performed in sealed glass containers (200 μL total volume in 12 mL glass tube) and then incubated at 37 °C with rotation at 60 rpm. After the reaction, the vessel was brought to room temperature, 1× volume of hexane was quickly added then the mixture was transferred to solvent resistant centrifuge tubes. The mixture was then vigorously vortexed and centrifuged, and the organic phase was collected for GC-FID.

### Gas-chromatography

For GC-FID, 1 μL of sample from the respective organic phases were injected automatically with an autosampler system, in split-less mode. Ultrapure helium was used as carrier with the flow set to 30 mL/min in constant flow mode. Separation was carried out on a Thermo-Scientific TG-WAXMS column with dimensions of 30 m × 320 μm × 0.25 μm. The Flame Ionization Detector was ignited with 350 mL/min ultrapure air and 35 mL/min hydrogen at constant flow. Data was recorded with Chromeleon 7 software by Thermo Fisher Scientific (Waltham, MA).

For acetone detection, the initial oven temperature was set to 35 °C for 2 min followed by a 10 °C/min ramp to 140 °C, then a second temperature ramp of 50 °C/min to a final temperature of 235 °C, which was maintained for 3 min. The Flame Ionization Detector was set at 250 °C.

For isoprenol detection, the initial oven temperature was set to 50 °C for 2 min followed by a 50 °C/min ramp to 80 °C, then a 5 °C/min ramp to 125 °C, then a ramp of 50 °C/min to a final temperature of 235 °C, which was then maintained for 3 min. The Flame Ionization Detector was set at 250 °C.

### ATPase contamination assay

To assay background ATPase activity, we used a coupled pyruvate kinase (PK) lactate dehydrogenase (LDH) assay. 5 μL of each enzyme was co-incubated with PK/LDH assay mix. A master mix was made fresh containing 3 mM NADH, 3 mM ATP, 3 mM phosphoenolpyruvate, and 3 μL PK/LDH mixture per 195 μL total (Sigma-Aldrich, St Louis, MO) buffered in General Buffer. To assay enzymes, 5 μL of enzyme sample was mixed with 195 μL master mix. In the presence of ATPase, ATP is hydrolyzed to ADP, triggering the PK/LDH reaction to consume NADH, which can be conveniently monitored by absorbance at 340 nm. Nox was not included in these assays since it directly consumes NADH.

## Supplementary Information

Supplementary Information.
