# Supplementary material for: Expanding the use of ethanol as a feedstock for cell-free synthetic biochemistry by implementing acetyl-CoA and ATP generating pathways
Source: Sci Rep. 2022 May 11;12:7700. doi: 10.1038/s41598-022-11653-3 (PMC9095697; doi:10.1038/s41598-022-11653-3)
Supplement: Supplementary file 1 — Supplementary Information. [file 41598_2022_11653_MOESM1_ESM.docx]

**Supplementary Information**

**Expanding the use of ethanol as a feedstock for cell-free synthetic biochemistry by implementing acetyl-CoA and ATP generating pathways**

Hongjiang Liu, Mark A. Arbing and James U. Bowie

Department of Chemistry and Biochemistry, Molecular Biology Institute, UCLA-DOE Institute for Genomics and Proteomics, University of California Los Angeles, Los Angeles CA

**Supplemental Figure 1. ATP production without acetone generation. (a)** Schematic of the partial system that converts ethanol to acetate with ATP generation. ATP is recycled in this scheme by adding glucose and hexokinase (Hex) to generate glucose-6-phosphate (G6P). **(b)** ATP generation over time, measured by the production of G6P. Orange and blue points reflect the full system with or without ethanol input, respectively. Data points are the average of biological triplicates and the error bars reflect the standard deviation.

**Supplemental Figure 2. Production of acetone without ATP co-generation (a)** Schematic of the partial system to test the ability to upcycle to ethanol to acetone with added acetate. (**b)** The amount of acetone production from ethanol and acetate co-feeding. The bar height is the average of biological triplicates and the error bar reflects the standard deviation.

**Supplemental figure 3. Schematic of possible Mevalonic acid to isoprenol modules.** There are three possible pathways for converting MVA into isoprenol. The natural biosynthetic pathways all converge on isopentenyl pyrophosphate (MVK, PMVK, PMDC) at the cost of 3 ATP. In theory, however, MVA could be directly decarboxylated to isoprenol, but no enzyme is known to catalyze this reaction to our knowledge. Future bioprospecting could potentially lead to a promiscuous PMDC capable of direct decarboxylation of MVA. An alternative pathway that directly decarboxylates M5P has been demonstrated using *Anaerolinea thermophila* PMDC (AtPMDC), saving one ATP. We used this pathway (bold arrows) since it requires less ATP.

**Supplemental figure 4. Isoprenol module optimization tests reveal that AP dominates the reaction rate.**  The isoprenol module was set up as described in Materials and Methods, with enzymes concentrations set at initial guesses. To optimize, the enzyme concentrations either halved (Low) or doubled (High). The only enzyme concentration that affected the rate was AP. As the other enzymes were at relatively low concentrations, we focused on optimizing the amount of AP as described in the main text.

**Supplemental Table 1. Enzymes used in this work**

| Enzyme Symbol | Name | Source | E.C. |
| --- | --- | --- | --- |
| ADH | Alcohol Dehydrogenase | *Geobacillus steaothermophilus* | 1.1.1.1 |
| ALDH | Acetaldehyde Dehydrogenase | *Thermus thermophilus* | 2.1.2.10 |
| Nox | NADH oxidase | *Lactobacillus brevis (PROSS engineered)^1^* | 1.6.3.4 |
| Thl | Thiolase | *Thermus thermophilus* | 2.3.1.9 |
| PTA | Phosphate Acetyltransferase | *Geobacillus steaothermophilus* | 2.3.1.8 |
| ACK | Acetate Kinase | *Geobacillus steaothermophilus* | 2.7.2.1 |
| AtoAD | Acetate CoA-transferase | *Escherichia coli* | 2.8.3.8 |
| ADC | Acetoacetate Decarboxylase | *Clostridium acetobutylicum* | 4.1.1.4 |
| MVK | Mevalonate Kinase | *methanococcus jannaschii* | 2.7.1.36 |
| PMDC | Phosphomevalonate Decarboxylase | *Anaerolinea thermophila* | 4.1.1.99 |
| AP^2^ | Acid Phosphatase | *Wheat* | 3.1.3.2 |
| Hex | Hexokinase | *Thermotoga maritima* | 2.7.1.1 |
| PMVK | Phosphomevalonate kinase | *Streptococcus pyogenes* | 2.7.4.2 |
| NudB | Dihydroneopterin triphosphate diphosphatase | *Escherichia coli* | 3.6.1.67 |

1. Liu, H., Bowie, J.U. Cell-free synthetic biochemistry upgrading of ethanol to 1,3 butanediol. *Sci Rep* **11,**9449 (2021).
2. Purchased from MP Biomedical, product #MFCD00131847

**The DNA and amino acid sequences for enzymes used in this work**

**ADH**

**ATGAAAGCTGCAGTTGTGGAACAATTTAAAAAGCCGTTACAAGTGAAAGAAGTGGAAAAACCTAAGATCTCATACGGGGAAGTATTAGTGCGCATCAAAGCGTGTGGGGTATGCCATACAGACTTGCATGCCGCACATGGCGACTGGCCTGTAAAGCCTAAACTGCCTCTCATTCCTGGCCATGAAGGCGTCGGTGTAATTGAAGAAGTAGGTCCTGGGGTAACACATTTAAAAGTTGGAGATCGCGTAGGTATCCCTTGGCTTTATTCGGCGTGCGGTCATTGTGACTATTGCTTAAGCGGACAAGAAACATTATGCGAACGTCAACAAAACGCTGGCTATTCCGTCGATGGTGGTTATGCTGAATATTGCCGTGCTGCAGCCGATTATGTCGTAAAAATTCCTGATAACTTATCGTTTGAAGAAGCCGCTCCAATCTTTTGCGCTGGTGTAACAACATATAAAGCGCTCAAAGTAACAGGCGCAAAACCAGGTGAATGGGTAGCCATTTACGGTATCGGCGGGCTTGGACATGTCGCAGTCCAATACGCAAAGGCGATGGGGTTAAACGTCGTTGCTGTCGATTTAGGTGATGAAAAACTTGAGCTTGCTAAACAACTTGGTGCAGATCTTGTCGTCAATCCGAAACATGATGATGCAGCACAATGGATAAAAGAAAAAGTGGGCGGTGTGCATGCGACTGTCGTCACAGCTGTTTCAAAAGCCGCGTTCGAATCAGCCTACAAATCCATTCGTCGCGGTGGTGCTTGCGTACTCGTCGGATTACCGCCGGAAGAAATACCTATTCCAATTTTCGATACAGTATTAAATGGAGTAAAAATTATTGGTTCTATCGTTGGTACGCGCAAAGACTTACAAGAGGCACTTCAATTTGCAGCAGAAGGAAAAGTAAAAACAATTGTCGAAGTGCAACCGCTTGAAAACATTAACGACGTATTCGATCGTATGTTAAAAGGGCAAATTAACGGCCGCGTCGTGTTAAAAGTAGATTAA**

MKAAVVEQFKKPLQVKEVEKPKISYGEVLVRIKACGVCHTDLHAAHGDWPVKPKLPLIPGHEGVGVIEEVGPGVTHLKVGDRVGIPWLYSACGHCDYCLSGQETLCERQQNAGYSVDGGYAEYCRAAADYVVKIPDNLSFEEAAPIFCAGVTTYKALKVTGAKPGEWVAIYGIGGLGHVAVQYAKAMGLNVVAVDLGDEKLELAKQLGADLVVNPKHDDAAQWIKEKVGGVHATVVTAVSKAAFESAYKSIRRGGACVLVGLPPEEIPIPIFDTVLNGVKIIGSIVGTRKDLQEALQFAAEGKVKTIVEVQPLENINDVFDRMLKGQINGRVVLKVD

**ALDH**

**ATGTCCGAAAGGGTTAAGGTAGCCATCCTGGGCTCCGGCAACATCGGGACGGACCTGATGTACAAGCTCCTGAAGAACCCGGGCCACATGGAGCTTGTGGCGGTGGTGGGGATAGACCCCAAGTCCGAGGGCCTGGCCCGGGCGCGGGCCTTAGGGTTAGAGGCGAGCCACGAAGGGATCGCCTACATCCTGGAGAGGCCGGAGATCAAGATCGTCTTTGACGCCACCAGCGCCAAGGCCCACGTGCGCCACGCCAAGCTCCTGAGGGAGGCGGGGAAGATCGCCATAGACCTCACGCCGGCGGCCCGGGGCCCTTACGTGGTGCCCCCGGTGAACCTGAAGGAACACCTGGACAAGGACAACGTGAACCTCATCACCTGCGGGGGGCAGGCCACCATCCCCCTGGTCTACGCGGTGCACCGGGTGGCCCCCGTGCTCTACGCGGAGATGGTCTCCACGGTGGCCTCCCGCTCCGCGGGCCCCGGCACCCGGCAGAACATCGACGAGTTCACCTTCACCACCGCCCGGGGCCTGGAGGCCATCGGGGGGGCCAAGAAGGGGAAGGCCATCATCATCCTGAACCCGGCGGAACCCCCCATCCTCATGACCAACACCGTGCGCTGCATCCCCGAGGACGAGGGCTTTGACCGGGAGGCCGTGGTGGCGAGCGTCCGGGCCATGGAGCGGGAGGTCCAGGCCTACGTGCCCGGCTACCGCCTGAAGGCGGACCCGGTGTTTGAGAGGCTTCCCACCCCCTGGGGGGAGCGCACCGTGGTCTCCATGCTCCTGGAGGTGGAGGGGGCGGGGGACTATTTGCCCAAATACGCCGGCAACCTGGACATCATGACGGCTTCTGCCCGGAGGGTGGGGGAGGTCTTCGCCCAGCACCTCCTGGGGAAGCCCGTGGAGGAGGTGGTGGCGTAA**

MSERVKVAILGSGNIGTDLMYKLLKNPGHMELVAVVGIDPKSEGLARARALGLEASHEGIAYILERPEIKIVFDATSAKAHVRHAKLLREAGKIAIDLTPAARGPYVVPPVNLKEHLDKDNVNLITCGGQATIPLVYAVHRVAPVLYAEMVSTVASRSAGPGTRQNIDEFTFTTARGLEAIGGAKKGKAIIILNPAEPPILMTNTVRCIPEDEGFDREAVVASVRAMEREVQAYVPGYRLKADPVFERLPTPWGERTVVSMLLEVEGAGDYLPKYAGNLDIMTASARRVGEVFAQHLLGKPVEEVVA

**Nox**

**ATGAAAGTTACAGTGGTTGGGTGTACGCACGCCGGCACGTTTGCAATTAAGCAAATTCTGAAAGAGCACCCAGACGCAGAAGTCACCGTCTACGAACGTAACGATGTGATCTCATTTCTGTCGTGCGGAATCGCCCTGTATTTAGGAGGGCAGGTGAAGGACCCACAAGGATTGTTTTATTCCTCGCCAGAGGAATTACAGAAACTTGGTGCCAATGTCCAAATGAATCATAATGTTTTGGCCATCGATCCGGATAACAAAACAGTTACCGTCGAAGATTTGACGAATGGGGAGCAGTTTACTGAAAGTTATGACAAGTTGGTAATGACATCTGGATCGTGGCCGATCGTGCCTAAAATCCCCGGTATCGACTCCGACCGCGTGCAGCTGTGTAAAAATTGGGCGCATGCCCAAGAGCTTTACGAGCGCGCAAAAGAGGCGAAGCGTATTGTCGTTATTGGAGCGGGCTATATCGGTGCAGAATTGGCCGAGGCTTATAGCACAACGGGGCATGATGTAACTCTGATTGATGCGATGGCGCGTGTCATGCCGAAATATTTCGACAAAGAATTCACTGACGTGATCGAACAGGACTACCGCGACCATGGTGTCCAGTTAGCACTTGGAGAGACAGTGGAATCGTTCGAGGATTCAGCCAATGGTCTGACTATTAAAACTGACAAGGGGTCTTATGAGACTGATTTAGCAATTCTTTGTATCGGGTTTCGCCCAAACACTGATTTATTAAAGGGCAAGGTGGATATGTTACCAAATGGTGCCATCATCACCGACGATTATATGCGCTCCTCCAACCCGGATATTTTCGCCGCTGGCGATTCTGCCGCCGTTCATTACAACCCTACTCATCAATATACTTACATCCCTCTTGCTACGAACGCTGTACGCCAGGGTATCCTGGTTGGGAAAAATTTGGTTAAGCCAACCGTGAAGTACATGGGCACACAGTCCAGCTCTGGATTAGCTCTGTACGATCGCACCATTGTAAGCACCGGCTTAACGCTGGAGGCCGCAAAACAACTTGGCTTAAACGCCGCGCAGGTGATTGTAGAAGATAATTACCGTCCTGAGTTTATGCCGACCACGGAGCCGGTGTTAATGTCCCTTGTGTACGATCCCGACACTCATCGCATCTTGGGTGGTCAGCTGATGTCTAAGTATGATGTCAGTCAGTCCGCCAACACTTTGTCGGTTTGCATCCAGAACAAAATGACAATTGACGACCTGGCCATGGTCGACATGCTTTTTCAGCCCAACTTTGATCGCCCTTGGAACTACCTTAATATTCTGGCGCAGGCTGCTCAAGCGAAGGTGGCGCAATCGGTAAACTAA**

MKVTVVGCTHAGTFAIKQILKEHPDAEVTVYERNDVISFLSCGIALYLGGQVKDPQGLFYSSPEELQKLGANVQMNHNVLAIDPDNKTVTVEDLTNGEQFTESYDKLVMTSGSWPIVPKIPGIDSDRVQLCKNWAHAQELYERAKEAKRIVVIGAGYIGAELAEAYSTTGHDVTLIDAMARVMPKYFDKEFTDVIEQDYRDHGVQLALGETVESFEDSANGLTIKTDKGSYETDLAILCIGFRPNTDLLKGKVDMLPNGAIITDDYMRSSNPDIFAAGDSAAVHYNPTHQYTYIPLATNAVRQGILVGKNLVKPTVKYMGTQSSSGLALYDRTIVSTGLTLEAAKQLGLNAAQVIVEDNYRPEFMPTTEPVLMSLVYDPDTHRILGGQLMSKYDVSQSANTLSVCIQNKMTIDDLAMVDMLFQPNFDRPWNYLNILAQAAQAKVAQSVN

**Thl**

**ATGCGTGAAGTGTATGTAGTCGCGGCAGTCCGTACCCCAATCGGCAAATTTGGCGGCGTATTCAAGGACGTTAGTCCCGTTGACTTAGGCGCGCACGCGATGCGTGAAGCATTAGCTCGTGCGGGGGTAGAGGGAAAGGCGTTGGATTTGTATATCTTCGGAAATGTTCTTCGCGCAGGACACGGCCAATTGTTACCGCGCCAAGCGGCGTTGAAGGCCGGTATCCCCAAAGAGGTCGACGGGTACCAAGTCGACATGGTGTGTGCCAGTGGCATGATGGCCGCGCTGAACGCGGTTCAATTCTTACGTACCGGCGAGGCCCATCTTGTGCTGGCCGGTGGGATGGAGTCAATGTCCCAAGCGGGTTTTTATCTTAGCCACCGTGCCCGTTGGGGGTATAAATTTTTACTTGGTGCACCCGAAAATCTTCAGGATATCCTGCTTCGTGATGGACTGTCGGACCCATTTACCGGCGAAGCGATGGGTGAGCAGGCCGAACGCTTAGCTCAGGATCATGGTGTGACTCGCCGCGAAATTGATGAAGCGGCCTATCTGAGTCACAAGCGCGCTGCGGAGGCCACTGAAAAGGGCCTTTTCGCGTGGGAGATTGCGCCGATGGAGGTGCAGGGTCGTAAAGGCCCCGTAGTCGTGGACCGCGACGAGGGAATCCGTCCTGAAACTACACTTGAGAGCTTGGCGGCTTTGCGCCCGGCGTTCAAAAAAGATGGAGTGCTGACCGCAGGAAATTCATCTCAGATCTCGGACGGCGCAGCGGCTTTGCTTCTTGCGTCAGAAGAAGCTGTGAAAGCCCACGGGCTTAAGCCAATTGCGAAGGTACTGGGGGGCGCGTGGGCTGCTGGGGAGTCATGGCGTTTCCCTGAAGCACCGATTCCGGCTGCTAAACGCTTACTGGACCGTTTAGGCATGCGCGTGTCTGATTTCGGGCTTTTCGAGAATAACGAAGCGTTCGCATTGAATAATGTCCTGTTTTCCCGTCTGCTGGATGTTCCCTACGAGCGCTTGAATGTGTTCGGTGGCGCTGTAGCCTTGGGACATCCTATTGGAGCGAGTGGAGCACGCATTTTGGTGACCTTACTGAATGCGTTACGTGCGAAGGGCGAGGAGCGCGGGCTTGCCGCAATCTGTCATGGTACAGGTGGTAGTGTTGCTTTCGCGGTTGAAGTAGTATAA**

MREVYVVAAVRTPIGKFGGVFKDVSPVDLGAHAMREALARAGVEGKALDLYVFGNVLRAGHGQLLPRQAALKAGIPKEVDGYQVDMVCASGMMAVLNAVQFLRTGEAHLVLAGGMESMSQAGFYLSHRARWGYKFLLGAPENLQDILLRDGLSDPFTGEAMGEQAERLAQDHGVTRQEIDEAAYLSHKRAAEATEKGLFAWEIAPMEVQGRKGPVVVDRDEGIRPETTLESLAALRPAFKKDGVLTAGNSSQISDGAAALLLASEEAVKAHGLKPLARVLGGAWAAGEPWRFPEAPIPAAKRLLDRLGMRVSDFGLFENNEAFALNNVLFSRLLDVPYERLNVFGGAVALGHPIGASGARILVTLLNALRAKGEERGLAAICHGTGGSVAFAVERV

**PTA**

**ATGACAACCGATTTATTTACGGCATTAAAAGCGAAAGTAACCGGTACGGCTCGAAAAATCGTGTTTCCCGAGGGAACCGATGACCGCATCTTAACGGCGGCGAGCCGTTTGGCGACGGAGCAAGTGCTTCAGCCGATCGTCCTTGGCGATGAGCAAGCGATAAGGGTGAAAGCAGCTGCGCTTGGCTTGCCGCTTGAAGGGGTGGAGATTGTCAACCCGCGCCGCTACGGCGGGTTTGATGAGCTAGTTTCGGCGTTTGTGGAGCGGCGCAAAGGGAAAGTGACAGAAGAAACGGCGCGCGAGTTGCTTTTCGATGAAAACTATTTCGGTACGATGCTCGTTTATATGGGAGCGGCCGACGGCCTCGTCAGCGGGGCGGCACATTCGACGGCGGATACGGTCCGACCAGCCTTGCAAATCATTAAAACGAAGCCAGGCGTTGACAAAACGTCCGGCGTGTTCATCATGGTGCGCGGCGACGAAAAATATGTGTTTGCCGATTGCGCCATCAACATTGCTCCTAACAGTCATGATTTGGCTGAAATCGCGGTCGAGAGCGCCCGGACGGCCAAAATGTTCGGCCTTAAGCCGCGCGTAGTGCTGTTAAGCTTTTCCACGAAAGGGTCGGCCTCGTCGCCGGAGACGGAAAAAGTCGTTGAGGCGGTGCGGTTGGCGAAAGAAATGGCGCCGGATCTGATCCTTGACGGTGAGTTTCAATTTGACGCCGCGTTTGTGCCAGAGGTGGCGAAAAAGAAAGCGCCGGACTCGGTCATTCAAGGGGACGCAAATGTCTTTATTTTCCCGAGCCTTGAGGCGGGCAACATCGGCTACAAAATCGCCCAGCGCCTTGGCGGCTTTGAAGCGGTTGGCCCGATTTTGCAAGGGCTGAACAAGCCGGTTAACGACCTATCGCGCGGCTGCAGCGCCGAAGACGCCTACAAGCTCGCGCTCATCACCGCGGCGCAGTCGCTTGGGGAGTAA**

MTTDLFTALKAKVTGTARKIVFPEGTDDRILTAASRLATEQVLQPIVLGDEQAIRVKAAALGLPLEGVEIVNPRRYGGFDELVSAFVERRKGKVTEETARELLFDENYFGTMLVYMGAADGLVSGAAHSTADTVRPALQIIKTKPGVGKTSGVFIMVRGDEKYVFADCAINIAPNSQDLAEIAVESARTAKMFGLKPRVALLSFSTKGSASSPETEKVVEAVRLAKEMAPDLILDGEFQFDAAFVPEVAKKKAPDSVIQGDANVFIFPSLEAGNIGYKIAQRLGGFEAVGPILQGLNKPVNDLSRGCSAEDAYKLALITAAQSLGE

**ACK** **ATGGCAAAAGTGTTAGCCGTTAATGCGGGAAGTTCTTCGTTGAAATTCCAATTGTTTGACATGCCGGCGGAAACGGTGTTAACGAAAGGAATCGTCGAGCGGATCGGCTTTGACGACGCGATTTTTACGATCGTCGTGAACGGGGAGAAACAGCGGGAAGTCACTTCCATCCCGAACCATGCCGTGGCGGTGAAACTGCTGCTTGACAAACTGATTCGCTATGGCATCATCCGGTCATTTGACGAAATTGACGGCATCGGCCATCGCGTCGTCCACGGCGGGGAGAAGTTCAGCGATTCGGTGTTGATCACCGATGAGGTGATAAAACAAATCGAAGAAGTGTCCGAGCTCGCTCCGCTTCATAACCCGGCCAACCTCGTCGGCATCCGCGCGTTTCAGGAAGTGCTGCCGAACGTGCCGGCCGTCGCCGTTTTTGATACGGCGTTTCACCAAACGATGCCGGAACAGTCGTTTTTGTACAGCTTGCCGTATGAGTATTACACGAAATTCGGCATTCGCAAGTACGGCTTCCATGGCACGTCGCACAAATACGTCACCCAGCGGGCGGCGGAGCTTCTCGGCCGGCCGATCGAGCAGCTGCGCCTCATCTCGTGCCATTTAGGCAACGGGGCGAGCATCGCGGCGGTCGAAGGCGGCAAATCGATCGACACGTCGATGGGCTTTACGCCATTAGCGGGCGTCGCGATGGGGACGCGCTCTGGCAACATCGACCCGGCGCTTATCCCATACATTATGGAAAAAACAGGAATGACCGTTAATGAAGTGATTGAAGTGCTGAATAAAAAGAGCGGCATGCTCGGCATCTCCGGCATCTCGAGCGACTTGCGCGACTTGGAAAAAGCGGCCGCCGAAGGAAATGAGCGCGCGGAACTTGCGTTGGAAGTGTTTGCGAACCGCATTCATAAATACATCGGCTCGTATGCGGCGCGCATGTGCGGCGTCGACGCCATCATTTTCACCGCCGGCATCGGCGAAAACAGCGAAGTCGTGCGGGCCAAAGTGTTGCGCGGCCTCGAGTTTATGGGAGTTTACTGGGATCCCATCCTAAACAAAGTGCGCGGCAAAGAAGCGTTCATCAGCTACCCGCACTCGCCGGTCAAAGTGCTCGTCATCCCGACGAACGAAGAGGTCATGATCGCCCGTGATGTCATGCGGCTGGCGAATTTGTAA**

MAKVLAVNAGSSSLKFQLFDMPAETVLTKGIVERIGFDDAIFTIVVNGEKQREVTSIPNHAVAVKLLLDKLIRYGIIRSFDEIDGIGHRVVHGGEKFSDSVLITDEVIKQIEEVSELAPLHNPANLVGIRAFQEVLPNVPAVAVFDTAFHQTMPEQSFLYSLPYEYYTKFGIRKYGFHGTSHKYVTQRAAELLGRPIEQLRLISCHLGNGASIAAVEGGKSIDTSMGFTPLAGVAMGTRSGNIDPALIPYIMEKTGMTVNEVIEVLNKKSGMLGISGISSDLRDLEKAAAEGNERAELALEVFANRIHKYIGSYAARMCGVDAIIFTAGIGENSEVVRAKVLRGLEFMGVYWDPILNKVRGKEAFISYPHSPVKVLVIPTNEEVMIARDVMRLANL

**AtoAD complex**

**ATGAAAACAAAATTGATGACATTACAAGACGCCACCGGCTTCTTTCGTGACGGCATGACCATCATGGTGGGCGGATTTATGGGGATTGGCACTCCATCCCGCCTGGTTGAAGCATTACTGGAATCTGGTGTTCGCGACCTGACATTGATAGCCAATGATACCGCGTTTGTTGATACCGGCATCGGTCCGCTCATCGTCAATGGTCGAGTCCGCAAAGTGATTGCTTCACATATCGGCACCAACCCGGAAACAGGTCGGCGCATGATATCTGGTGAGATGGACGTCGTTCTGGTGCCGCAAGGTACGCTAATCGAGCAAATTCGCTGTGGTGGAGCTGGACTTGGTGGTTTTCTCACCCCAACGGGTGTCGGCACCGTCGTAGAGGAAGGCAAACAGACACTGACACTCGACGGTAAAACCTGGCTGCTCGAACGCCCACTGCGCGCCGACCTGGCGCTAATTCGCGCTCATCGTTGCGACACACTTGGCAACCTGACCTATCAACTTAGCGCCCGCAACTTTAACCCCCTGATAGCCCTTGCGGCTGATATCACGCTGGTAGAGCCAGATGAACTGGTCGAAACCGGCGAGCTGCAACCTGACCATATTGTCACCCCTGGTGCCGTTATCGACCACATCATCGTTTCACAGGAGAGCAAATAA*TGGATGCGAAACAACGTATTGCGCGCCGTGTGGCGCAAGAGCTTCGTGATGGTGACATCGTTAACTTAGGGATCGGTTTACCCACA*ATGGTCGCCAATTATTTACCGGAGGGTATTCATATCACTCTGCAATCGGAAAACGGCTTCCTCGGTTTAGGCCCGGTCACGACAGCGCATCCAGATCTGGTGAACGCTGGCGGGCAACCGTGCGGTGTTTTACCCGGTGCAGCCATGTTTGATAGCGCCATGTCATTTGCGCTAATCCGTGGCGGTCATATTGATGCCTGCGTGCTCGGCGGTTTGCAAGTAGACGAAGAAGCAAACCTCGCGAACTGGGTAGTGCCTGGGAAAATGGTGCCCGGTATGGGTGGCGCGATGGATCTGGTGACCGGGTCGCGCAAAGTGATCATCGCCATGGAACATTGCGCCAAAGATGGTTCAGCAAAAATTTTGCGCCGCTGCACCATGCCACTCACTGCGCAACATGCGGTGCATATGCTGGTTACTGAACTGGCTGTCTTTCGTTTTATTGACGGCAAAATGTGGCTCACCGAAATTGCCGACGGGTGTGATTTAGCCACCGTGCGTGCCAAAACAGAAGCTCGGTTTGAAGTCGCCGCCGATCTGAATACGCAACGGGGTGATTTATGA (italic blue region is the linker sequence)**

**AtoD:** MKTKLMTLQDATGFFRDGMTIMVGGFMGIGTPSRLVEALLESGVRDLTLIANDTAFVDTGIGPLIVNGRVRKVIASHIGTNPETGRRMISGEMDVVLVPQGTLIEQIRCGGAGLGGFLTPTGVGTVVEEGKQTLTLDGKTWLLERPLRADLALIRAHRCDTLGNLTYQLSARNFNPLIALAADITLVEPDELVETGELQPDHIVTPGAVIDHIIVSQESK

**AtoA:**

MVANYLPEGIHITLQSENGFLGLGPVTTAHPDLVNAGGQPCGVLPGAAMFDSAMSFALIRGGHIDACVLGGLQVDEEANLANWVVPGKMVPGMGGAMDLVTGSRKVIIAMEHCAKDGSAKILRRCTMPLTAQHAVHMLVTELAVFRFIDGKMWLTEIADGCDLATVRAKTEARFEVAADLNTQRGDL

**ADC**

**ATGCTCAAAGATGAAGTTATTAAGCAGATCAGTACCCCGTTAACCTCTCCGGCATTCCCACGTGGCCCTTACAAATTCCATAACCGTGAATACTTTAACATCGTTTATCGTACTGATATGGACGCCCTGCGCAAAGTTGTGCCCGAACCCCTCGAGATCGATGAACCGCTCGTGCGCTTTGAAATTATGGCAATGCATGATACCTCAGGGCTGGGATGTTATACGGAGTCTGGTCAGGCCATTCCGGTCAGCTTCAATGGGGTAAAAGGGGATTACCTCCATATGATGTACCTCGATAATGAACCGGCAATTGCGGTGGGCCGCGAACTGAGCGCATATCCGAAAAAACTGGGGTATCCGAAACTGTTCGTTGACAGCGATACGTTGGTTGGCACCCTCGATTATGGAAAACTGCGCGTGGCGACTGCTACCATGGGGTACAAGCACAAAGCCTTGGACGCGAACGAGGCGAAGGACCAAATTTGCCGTCCTAACTATATGCTTAAGATTATTCCCAATTATGATGGATCTCCGCGTATTTGCGAACTCATTAATGCCAAAATTACCGACGTTACGGTTCATGAGGCATGGACAGGCCCGACCCGCTTGCAACTGTTTGACCACGCCATGGCCCCGCTGAATGATTTACCGGTAAAAGAAATTGTATCCAGTAGCCATATCTTGGCCGATATTATCTTGCCGCGCGCGGAAGTTATTTACGATTATCTGAAATAA**

MLKDEVIKQISTPLTSPAFPRGPYKFHNREYFNIVYRTDMDALRKVVPEPLEIDEPLVRFEIMAMHDTSGLGCYTESGQAIPVSFNGVKGDYLHMMYLDNEPAIAVGRELSAYPKKLGYPKLFVDSDTLVGTLDYGKLRVATATMGYKHKALDANEAKDQICRPNYMLKIIPNYDGSPRICELINAKITDVTVHEAWTGPTRLQLFDHAMAPLNDLPVKEIVSSSHILADIILPRAEVIYDYLK

**MVK**

**ATGATTATTGAAACCCCATCCAAAGTTATCTTATTCGGGGAGCACGCAGTAGTATATGGGTACCGCGCAATTTCAATGGCTATTGATTTAACGTCAACGATCGAGATTAAGGAGACGCAGGAAGACGAAATCATCCTTAATCTGAACGACCTGAACAAAAGTCTGGGCTTGAATTTGAACGAGATCAAGAATATCAATCCAAACAATTTCGGGGACTTTAAGTACTGTCTGTGCGCTATTAAAAATACCTTAGATTACCTTAATATCGAGCCTAAGACCGGCTTCAAAATTAACATCTCTTCGAAGATTCCCATCTCGTGCGGGCTTGGTTCCAGTGCGTCGATTACCATCGGAACTATCAAAGCTGTCAGCGGCTTTTACAACAAAGAATTGAAAGATGATGAAATTGCAAAATTAGGCTACATGGTAGAAAAGGAGATCCAAGGGAAAGCTAGTATCACCGACACGAGCACCATCACTTACAAGGGGATTTTGGAGATTAAGAACAATAAGTTTCGCAAAATTAAAGGCGAATTTGAAGAGTTCCTGAAGAACTGCAAATTCTTAATCGTTTACGCAGAGAAGCGTAAGAAGAAGACCGCCGAGCTTGTAAATGAGGTTGCGAAAATTGAAAATAAGGACGAGATCTTCAAAGAGATTGATAAGGTCATTGACGAGGCTCTGAAGATTAAAAACAAGGAAGACTTCGGAAAACTTATGACAAAAAATCATGAGTTACTTAAAAAGTTGAACATCAGTACCCCAAAGCTGGACCGCATCGTAGATATTGGAAACCGTTTTGGATTCGGGGCGAAATTAACCGGAGCGGGAGGAGGTGGTTGCGTCATTATTTTAGTTAATGAAGAGAAGGAGAAGGAGCTGCTTAAGGAGCTGAATAAGGAGGATGTTCGTATTTTCAACTGCCNTATGANGAATTAA**

MIIETPSKVILFGEHAVVYGYRAISMAIDLTSTIEIKETQEDEIILNLNDLNKSLGLNLNEIKNINPNNFGDFKYCLCAIKNTLDYLNIEPKTGFKINISSKIPISCGLGSSASITIGTIKAVSGFYNKELKDDEIAKLGYMVEKEIQGKASITDTSTITYKGILEIKNNKFRKIKGEFEEFLKNCKFLIVYAEKRKKKTAELVNEVAKIENKDEIFKEIDKVIDEALKIKNKEDFGKLMTKNHELLKKLNISTPKLDRIVDIGNRFGFGAKLTGAGGGGCVIILVNEEKEKELLKELNKEDVRIFNCRMMN

**PMDC**

**ATGGGCCAGGCGACCGCCATCGCGCATCCGAACATTGCTTTCATTAAATATTGGGGTAATCGCGACGCCGTTCTTCGTATTCCGGAAAATGGCAGTATTTCAATGAATCTGGCCGAACTGACTGTAAAAACCACGGTTATTTTTGAAAAACATTCTCGTGAAGATACGCTGATCTTAAACGGCGCTCTGGCGGATGAACCGGCGCTGAAACGCGTTTCGCACTTTCTTGATCGCGTACGCGAGTTTGCAGGCATTTCGTGGCACGCACATGTTATTAGCGAGAACAACTTTCCGACTGGGGCCGGTATCGCGTCAAGCGCCGCCGCCTTTGCCGCGCTCGCGCTGGCCGCGACCTCTGCTATTGGTCTGCATTTAAGTGAACGTGATCTGAGCCGCCTCGCCCGCAAAGGGTCCGGCTCGGCGTGTCGCTCAATTCCTGGCGGTTTCGTCGAGTGGATCCCGGGCGAGACGGATGAAGATTCCTATGCAGTGTCGATCGCCCCGCCCGAACATTGGGCCCTGACCGATTGCATTGCGATTTTGAGTACCCAGCATAAACCCATCGGTTCTACTCAGGGTCATGCACTGGCCAGTACATCCCCCCTGCAGCCGGCGCGCGTGGCGGATACTCCTCGTCGGCTGGAAATTGTCCGTCGTGCGATTCTCGAACGTGATTTTCTGTCGCTCGCCGAAATGATCGAACATGATTCCAACCTGATGCACGCAGTTATGATGACGTCAACCCCGCCCTTATTTTATTGGGAACCGGTGAGCCTTGTAATCATGAAATCTGTGCGCGAATGGCGCGAATCTGGTCTCCCTTGCGCCTACACTTTGGATGCCGGTCCGAACGTGCACGTGATTTGCCCCTCCGAATATGCAGAGGAAGTGATTTTTCGGCTGACGAGCATTCCAGGTGTGCAAACGGTCCTTAAGGCCTCTGCTGGTGATTCAGCCAAGCTGATCGAGCAGTCCCTGTAA**

MGQATAIAHPNIAFIKYWGNRDAVLRIPENGSISMNLAELTVKTTVIFEKHSREDTLILNGALADEPALKRVSHFLDRVREFAGISWHAHVISENNFPTGAGIASSAAAFAALALAATSAIGLHLSERDLSRLARKGSGSACRSIPGGFVEWIPGETDEDSYAVSIAPPEHWALTDCIAILSTQHKPIGSTQGHALASTSPLQPARVADTPRRLEIVRRAILERDFLSLAEMIEHDSNLMHAVMMTSTPPLFYWEPVSLVIMKSVREWRESGLPCAYTLDAGPNVHVICPSEYAEEVIFRLTSIPGVQTVLKASAGDSAKLIEQSL

**Hex**

**ATGCCAAAATTAAAATTAATCGGTGTTGACTTAGGTGGAACTACTTTCAGCGTTGGGCTTGTTAGCGAAGATGGTAAAATTCTGAAGAAAGTTACGCGCGACACGTTGGTCGAAAACGGAAAGGAGGATGTTATTCGTCGTATTGCAGAAACGATCTTAGAAGTATCCGATGGAGAGGAGGCGCCTTACGTGGGAATTGGGAGTCCGGGCAGCATCGACCGCGAAAACGGCATCGTTCGTTTCAGCCCAAATTTCCCTGATTGGCATAATGTGCCCCTGACTGACGAACTGGCTAAGCGTACAGGGAAAAAGGTATTTCTGGAAAACGATGCTAATGCCTTTGTGCTTGGGGAAAAATGGTTTGGAGCCGGACGTGGTCACGATCACATCGTTGCGCTGACGCTTGGGACAGGGATCGGAGGTGGGGTTGTAACTCACGGATACTTGTTGACGGGGCGTGACGGGATTGGTGCAGAGTTGGGACACGTCGTGGTGGAGCCTAATGGACCTATGTGTAACTGTGGGACGCGCGGATGTCTGGAGGCTGTCGCTTCAGCCACTGCAATCCGCCGCTTTCTGCGCGAGGGATATAAAAAGTACCATTCTTCATTAGTCTATAAGCTTGCAGGTAGCCCCGAGAAGGCTGACGCGAAGCACTTGTTTGACGCGGCGCGTCAAGGGGATCGTTTTGCGCTGATGATCCGTGACCGCGTTGTTGACGCTTTGGCTCGCGCCGTTGCAGGTTATATTCATATCTTTAACCCCGAAATCGTTATTATTGGAGGTGGCATTTCACGTGCTGGAGAGATTTTGTTCGGTCCCCTTCGCGAAAAGGTCGTTGACTATATTATGCCCAGTTTCGTAGGGACGTATGAGGTGGTGGCCTCACCATTGGTAGAGGACGCGGGTATTTTGGGAGCGGCCTCGATCATTAAGGAGCGTATTGGCGCGTAA**

MPKLKLIGVDLGGTTFSVGLVSEDGKILKKVTRDTLVENGKEDVIRRIAETILEVSDGEEAPYVGIGSPGSIDRENGIVRFSPNFPDWHNVPLTDELAKRTGKKVFLENDANAFVLGEKWFGAGRGHDHIVALTLGTGIGGGVVTHGYLLTGRDGIGAELGHVVVEPNGPMCNCGTRGCLEAVASATAIRRFLREGYKKYHSSLVYKLAGSPEKADAKHLFDAARQGDRFALMIRDRVVDALARAVAGYIHIFNPEIVIIGGGISRAGEILFGPLREKVVDYIMPSFVGTYEVVASPLVEDAGILGAASIIKERIGG
